# Monthly Review of Parisian Medicine and Surgery

**Published:** 1859-06

**Authors:** Theophilus Mack

**Affiliations:** Lecturer on Materia Medica and Therapeutics in the Medical Department of the University of Buffalo


ART IV.—*Monthly Review of Parisian Medicine and Surgery.* By THEOPHILUS MACK, M. D., Lecturer on Materia Medica and Therapeutics in the Medical Department of the University of Buffalo.

*Academy of the Sciences.*—M. Flourens describes, under the name of the "*Genito-Spinal Centre,*" a new centre of movement in the medulla spinalis, which occupies a space only of about a few lines. In the rabbit, this space is at the fourth lumbar vertebra, and gives origin to the fourth lumbar nerve, and a lumbar portion of the sympathetic nerve, giving involuntary motion to the inferior part of the intestinal canal, the bladder, and the vasa deferentia, which thus arises from the spinal cord and not in the ganglia. M. Flourens, also, on behalf of M. de Luca, of Naples, presented a case of ulcer of the stomach, cured by lime water.

M. Kuhne, who has been engaged in experiments upon the action of mineral irritants on the nerves and muscles, has communicated, at the meeting of March 7th, a series of new experiments with organic substances, such as are known to act upon the matter composing nerve and muscle. In the organism he has found one substance which has a most remarkable influence upon the muscles, and upon the nerves,—viz., bile. One drop of this secretion causes the muscles to contract and form a very characteristic mass. He also states that the muscles have an irritability independent of the nerves.

M. Jacubowitsch has recognized and described three particular forms of nerve cells, in relation one with the other, and in connection with three different orders of nervous fibres. He has determined the exact disposition of these diverse histological nervous elements of the medulla spinalis, medulla oblongata, and the brain; he has pointed out the points of the nervous centres wherein these cellules or fibres are grouped, accumulated, mingled, separate, appear, or disappear. This important work has been honored by the decree of the prize of experimental physiology.

M. Baillarger, on 23d Nov. ult., presented the brain of a woman forty-six years of age, who had suffered from congestive mania, with ambitious delusions. On the middle lobe, (right hemisphere) there was a gangrenous patch about one inch square, brownish, soft, and emitting a very fœtid smell. M. Baillarger thinks that the gangrene may be owing to the obliteration of some vessels.

The "*Abeille Médicale*" relates a somewhat remarkable case of tubage of the larynx. A child at Nantes, laboring under croup, was given up by the physician. The child's grandmother took a long goose-quill, dipped its feathers into spirits, thrust it into the child's windpipe, and actually cleaned it out, bringing away a large quantity of false membrane. Recovery ensued.

Two successful cases of tubage of the larynx, with instillations of solutions of tannin and of nitrate of silver are given by M. Loiseau, making in all thirty instances of cure by this mode of procedure. On the other hand, many observations of failure have been received.

In the columns of this journal, it will be remembered that Dr. Hunt reported a case of radical cure of spermatorrhœa by castration, the operation having been performed by some luminary very far *west*. This brilliant exploit has now been eclipsed by no less a personage than a London Hospital Physician; for we learn from a communication in the "*Gazette Médicale,*" that an American denizen having crossed the Atlantic to procure a cure for epilepsy, by tracheotomy, or if he could find any operator bold enough, by the *ablation of both testicles,* (he was a widower!) met with a man after his own heart. Mr. Holthouse, of the Westminster Hospital, completely castrated him. It is to be hoped the cure may be radical; as to the justifiability of the transaction, that is on a par with the first mentioned case. The poor patient, by the previous effects of nitrate of silver, had completely qualified himself for what he will probably be restored to us as—a black eunuch! perhaps epileptic withal. In fact, his epilepsy returns just as frequently:—his body has therefore gained but little at the hands of his metropolitan Chiron; mentally, we may infer, he enjoys all the serene dignity of castration.

The effects of arsenical green pigments in the dust from wall paper, upon the occupants of chambers so papered, and upon artizans employing the same, have been represented as injurious to the health of those parties, and some hygienists demand legal interference for the prevention of such slow internal and external poisoning.

In the town of Zablagen of Wurtemburg, a successful experiment of a highly philanthropic nature calls for especial imitation.

"M. Helgerad has assembled one hundred and sixty workmen, all deaf mutes; he has taught them the business of compositors, and at this day, this silent printing establishment works to a marvel. We cannot applaud too highly the idea which M. Helgerad has had, to make thus useful these unfortunates so cruelly treated by nature; the King of Wurtemburg has recompensed this good innovation by a gold medal awarded to the chief of the '*Imprimerie des Sourds-muets.*'"

We read in the "*Revue de Thérapeutique du Midi,*" a case of intestinal perforation by two lumbrici. "The lumbrici had attached themselves to the intestine (the ileum), had gnawed it, perforated it, and after engaging themselves in the opening which they had made, had penetrated into the abdominal cavity" at the moment of a strong exertion of the abdominal muscles, producing death.

"A young Mahometan woman, eight months after having made the pilgrimage to Mecca, passing by Medina, felt suddenly, one morning, a sharp pain, accompanied by insupportable itching in the inferior members, especially at the interior of the legs and at the popliteal space. At the same time she perceived that these parts were the seat of hard, red, burning tumors. The following evening some blisters which had formed, burst and gave exit to an abundant dark red, sanious liquid, and the patient discovered, hanging out, the extremity of a filiform body resembling a violin string, which she recognized from having seen it before.

"After fruitless efforts, she presented herself to M. Marc Ficipio, Professor in the imperial school of medicine of Constantinople, who found the existence of several hair-worms appearing at the bottom of sanious inflamed ulcers, and resulting from the tearing of the blisters. He fixed one of them upon a split stick, rolled it upon it and attached it upon the leg. After different accidents the others were seized in the same manner, and the patient was charged to wind them up each day.

"At the end of six weeks, six hair-worms had been extracted, four from the left leg, and two from the right. The cicatrization of the wounds had commenced, when a new Dracunculus appeared at the lower part of the right leg. It was seized like the others, and its extraction was followed by a complete cure."—*Gaz. Méd. d' Orient.*

The Editor of the "*Gaz. des Hôpitaux*," in referring to the case of embolus of the pulmonary vein, published in our last article, cites several cases of a similar nature, and adds that this accident is not confined to the puerperal state, but that "The obliteration of the pulmonary artery by fibrous clots, without primitive alteration of the arterial walls, has been observed at the end of the most different affections, every time that they were complicated with obliterating phlebitis, or with the obliteration of a vein by clots without phlebitis." Thus, in two cases reported in the work of Chariot and Benj. Bell, the primitive affection had been a phlebitis; in a third observation, death had been seen to come on suddenly in a female attacked with venous obliteration consecutive to a cancerous affection of the stomach and of the pancreas. Two observations of M. Virchow confirm a fact already pointed out by Pofessor Magnus Huss; namely, the spontaneous coägulation of the blood in the veins of the members, and the consecutive obliteration of the pulmonary artery by the clots at the termination of typhoid fever.

These examples, although relating to facts happily very rare, are yet numerous enough always to justify this conclusion drawn from their researches by M. Chariot and Bell; viz., that sudden or rapid death by fibrinous obliteration of the pulmonary artery, may happen, not only in the case of phlegmasia dolens, but also in the different cases where fibrinous coagula form in the principal veins in the limbs.

Doctor Voltolini presents to medical jurists a new sign of alcoholic poisoning. In cases of this description, the aortic and pulmonary valves assume a red cinnabar color, which gives a very beautiful appearance to the valves, and which cannot be effaced. A remarkable quantity of a dark fluid blood fills the ascending cava and subclavian veins.

Chlorinated water, as a lotion, is represented by a contributor to the "*Gaz. des Hôpitaux*," as an efficient prophylactic in dissection wounds.

A new diagnostic sign derived from the property which the metals possess applied to the surface of the body to exert a direct action upon the nervous functions and upon the muscular functions which depend upon them, has been communicated by Dr. Burg. The following practical application by M. Trousseau has been made of this aid in diagnosis.

A vigorous, healthy woman had her menses arrested by a sudden fright; this was succeeded by a violent delirium of two days duration; after recovering from which, she was found hemiplegic in an unequal degree upon the right side; this latter feature and hyperæsthesia of the vertex left M. Trousseau somewhat in doubt as to whether he had to deal with a paralysis resulting from congestion following menstrual suppression, or a simple nervous paralysis, when the experience of Dr. Burg came to his aid.

In the therapeutic application of the metals against neurosis, the following fact had been frequently observed :—in idiopathic paralysis or paralyses, purely nervous, when a metal, copper, brass, iron, gold, &c., was applied to any part of the skin, most ordinarily, the sensibility was seen to appear. This sensibility returned to the spot where the metal was applied, next to the neighboring parts, and a little after, muscular power returned.

On account of the suppression of the menses, M. Trousseau ordered bleeding, and Dr. Burg applied to the paralysed arm, one of his metallic armatures, and in a short period by hyperæs, thesia, supplanted the anæsthesia at the point where the metal was applied, thus establishing the purely nervous nature of the affection.

The originator claims great importance for this method of diagnosis in distinguishing between chlorosis and anæmia.

Any method of instantaneous cure merits our earnest attention on account of its extreme rarity in practical medicine; for this reason, we will give more *in extenso* a translation of an article in the "*Bulletin de Thérap.* from the pen of M. Briquet, upon the cure of two forms of rheumatism by "faradisation."

The first is a variety of megrim often called irisalgia; it follows exposure to the rays of the sun, and is attended by a category of painful symptoms. Currents of galvanism are sent across the affected muscles by moist sponges for about five minutes, to relieve the pain, and its return may be prevented by wearing blue spectacles upon first going out, which may be removed after about half an hour.

The second kind of muscular rheumatism is one considered a species of headache, and very common.

"Many lymphatic persons with fair, slightly animated skin, subject to rheumatic pains, frequently contracting coryza, contracting catarrh very easily, very sensitive of cold, have, upon rising in the morning, the head heavy, a dull pain embarrassing the movements of their neck; it seems to them as if a cap of lead covered their head; if we press upon the neck, the parts about the ears, the temples and the forehead, we give rise to a certain pain in the spot pressed upon.

"These ills sadden, give bad humor, create incapacity for movement, occasion repugnance to every kind of labor, and preöccupy much the parties attacked, because they never know if their indisposition will be dissipated in the day, or if, on the contrary, they are destined to suffer long. Often enough the cares of the toilette remove slowly this state of suffering; but often, also, this painful state continues all day: there are even cases with whom it is continual, and with whom life becomes insupportable. I have seen ladies become sad, morose, and in some sort hypochondriac, under the influence of this continual suffering.

"Nothing is more easy to make cease than this condition, which depends upon a simple rheumatism of the muscles of the neck; for the pains of the head are only an extension of it; it is enough to faradise the muscles of the neck, be it in making the current pass by the aid of the sponges across the muscles, if the pain be not too strong, perhaps by acting upon the skin with the aid of the metallic brush, if the rheumatism is intense or if it be tenacious."

The prophylaxis of this affection is very simple. He deems its cause to be, the forced extension of the fibres of the muscles of the neck by the position of the head during sleep. Upon going to bed, a species of bolster is placed under the neck, or a person arranges himself so that the pillow, not exceeding the height of the neck, fits well to this part, and leaves the head free to arrange itself behind; in this way the splenii, complexi, and trapezii are no longer tense, and in the morning the head is free and completely exempt from the weight so painful.

With respect to inhalations in the treatment of chronic diseases of the chest, M. Champouillon expresses himself to the following effect:

By inhalation of mineral waters and other medicated liquids, mineral substances are rudely and for a short time in action upon the pulmonary tissue; by digestion, they arrive slowly and in a continuous manner to the seat of the evil, which certainly does not diminish their efficacy. The lung easily takes offence at the contact of that which is not pure air; on the contrary, the stomach has an inexhaustible tolerance for all we desire to commit to it. "Let everything then remain in the state for which nature has created it."

The following causes are given for softening of the brain, by Professor Teipier (*Gaz. Méd. de Lyon.*)

"Grief resulting from unsuccessful ambition; losses at the gaming-table; political enthusiasm; abuse of all enjoyments of life; venereal excesses; inveterate syphilis; specific treatment carried on too long; literary pursuits accompanied by too great tension of the intellect; excessive use of spirits, especially of the spirit scented with wormwood; and lastly, the habit of *smoking a great deal of tobacco*."

Pernicious intermittent of the apoplectic forms is known in paludal regions in France, and very justly dreaded. Two fatal cases of this kind have occurred in my own practice, and have left no very pleasing impressions as to diagnosis, prognosis or treatment. Dr. Léon Sorbets describes it very faithfully in the "*Gazette des Hôpitaux*," and I will translate it, as I dare say many other practitioners will recognize the disease as being American as well as Gallic:

"Pernicious intermittent in our country places affects many forms. At one time it shows itself with serious symptoms of pleuro-pneumonia;. in this case it takes the name of pneumonic pernicious fever. At other times we observe vomiting and abundant diarrhœal flux. These phenomena are followed by weakness or absence of pulse, and lowering of animal temperature: to this set of symptoms we give the name of choleriform pernicious fever. Lastly, there is a third variety, the most grave of all perhaps, because it may pass unperceived, and because the expectation of it is impossible: it is the pernicious intermittent fever in the apoplectic form.

"In this last case, the scene opens by serious symptoms of cerebral congestion, with loss of intelligence and speech, and coldness of the extremities. The patient falls as if struck with a blow of a club. There are profound coma or general convulsions, stertorous breathing, and alteration of the features. If a prompt and immediate succor, an energetic antiphlogistic treatment for example, does not come to avert the cerebral congestion, this latter will convert itself into apoplexy. The cerebral hœmorrhage will give rise then to the phenomena of paralysis, varying according to the seat of the cerebral sanguine extravasation. In the contrary case, after one or two bleedings made in rapid succession, we may ordinarily witness the absence of symptoms of paralysis, the affection remaining limited to a simple cerebral congestion. But commonly, and while the congestive symptoms exist, other symptoms present themselves, which, although transient and difficult to recognize, no less exist. Irregular chills, yawnings, stretchings, uncomfortableness, with general coldness; a sharp headache, which adds more to the gravity of the symptoms of cerebral congestion ; all giving to this collection of phenomena a particular stamp, a physiognomy quite special. The physician, witness of this scene, may at first view diagnosticate an attack of apoplexy. But his examination should not stop at this first appearance ; he should see there another thing than a cerebral congestion, if there be during some weeks, in the country where he makes his observation, an epidemic of intermittent fevers, and above all, if the patient, after the ordinary antiphlogistic treatment, does not present any of the accidents of paralysis. If he complains of wandering chills and yawnings, he ought to hasten to the employment of the principal antiperiodics, the sulphate of Quinine in large doses, to hinder the reäppearance, the repetition of the same accidents, because if the treatment directed against the apoplexy has prevented the development of its ordinary symptoms, the pernicious fever, showing itself anew with its frightful attendants, might rapidly carry off the patient."

*Imperial Academy of Medicine.*—The centralization at Paris of this august body in solemn conclave, surrounded by a halo of imperial authority, is another instance of the happy genius for organization of the French nation ; thus employing the combined talent of the leaders of the profession in sifting novel statements, in debating old theories, or deducing principles from observed facts. Many difficulties are solved, modest merit educed, and much accomplished for the good of the healing art in general, by the wise discussions and prudent deliberations of so distinguished a body of medical *savants.*

M. Sappey read a report upon the way by which the blood of the vena porta is brought back into the vena cava inferior, when it can no more find a free passage through the liver. The existence of such a derivative current, considered as a morbid symptom, and evincing by the fact the presence of an ancient and incurable cirrhosis, should be considered, according to M. Sappey, more as a favorable sign, because it avoids the danger of dropsy.

M. Huguier read a memoir upon elongation of the neck of the uterus, tending to modify profoundly the most generally admitted opinions upon a whole class of lesions of the womb. He says :

" Falling of the uterus, complete or incomplete, is not a single malady, but rather an assemblage of many affections designated by a single name.

" When the uterus appears without, even when the vagina is completely inverted, and when the womb, by the volume of the tumor at the centre of which it is found, seems entirely precipitated between the thighs, it is not, in the great majority of cases, because it is lowered, wholly and completely departed from the pelvis, but rather because it has undergone a partial or general hypertrophic elongation.

"In the affection designated under the name of precipitation (procidentia), the hypertrophied lengthening is not the exception, but rather the very general rule."

The author goes on to describe two principal varieties of "longitudinal hypertrophy," the above and below the vagina.

The following is the summary of his ably written work :

"In the first species of elongation, the neck of the womb forms, in the cavity of the vagina, a cylindroid or conoid protuberance more or less lengthened, whose free extremity approaches the vulvar opening, or even engages itself between the lips of the part, without the vulvo-uterine conduit being contracted, invaginated or inverted upon itself.

"It has been until lately confounded with falling and descent of the uterus, when it has not been taken and treated for a polypus, a chronic inversion, a follicular cyst or scirrhus of the neck, or a dropsy of that part.

"No complete description has yet been written, although it has well defined characters in relation to its development, its symptoms and its treatment.

"Medical measures and the different kinds of cauterization are not applicable except to slight hypertrophies, and to those complicated with inflammation and engorgement.

"Pessaries are most frequently useless or dangerous.

"When the elongation reaches five to seven centimetres, there is but one truly effectual and curative method to employ : this is the resection of the neck at half a centimetre below the insertion of the vagina."

"The disease called prolapsus uteri has received no semeiotic or anatomo-pathological proof; on the contrary, our observations prove the frequency of longitudinal hypertrophy.

In the treatment of this affection, we should not have recourse to a surgical operation except when it produces serious accidents, and when we have a certitude that medical and milder local measures are "insufficient."

He insists upon the amputation close to the body of the organ.

"This operation should never be practised before having taken preliminary cautions against subsequent inflammations. These precautions should be continued with the greatest care during the fifteen or twenty days which follow.

"The arteries of the uterine tissue are very difficult to seize and to tie ; to achieve this promptly and safely, we must make use of a species of tenaculum, which we allow to remain until it falls off spontaneously.

"The ecraseur has appeared to us useful to finish the section of the neck, above all, if this part is very vascular."

He states that he has succeeded, after resection of the neck, in operating for cystocele and rectocele, previously complicating the disease. He considers the operation contra-indicated when there is coëxistent a large pelvis and very large vulvar aperture, a torn perineum and excessive weakness of all the soft parts which form the floor of the pelvis.

"When we do not operate in the conditions pointed out, the malady does not return, and the health becomes as flourishing as it was before the development of the affection."

It is evident M. Huguier has exaggerated this bantling of his, although his work abounds in merit of the highest order. His propositions were contested ably, and M. Depaul closed his remarks by declaring, "that amputation of the uterine neck is a very serious operation, which may compromise the life of the patients, perhaps by peritonitis, perhaps by menorrhagia; that it must be rarely had recourse to, and not practised in cases of absolute necessity, until after having exhausted the series of means proposed against hypertrophy of the neck of the womb."

At the Society of Practical Medicine, M. Mattei read a work entitled, "Albuminuria in general, and more especially during pregnancy. Of eclampsia and of premature delivery as a measure to suspend the eclampsic convulsions."

In the wards under the charge of M. Chassaignac, drainage tubes are made use of for the purpose of keeping up a constant free discharge from suppurating cavities. These are fine caoutchouc tubes, having the two ends tied, which are passed through abscesses or sinuses leading to dead bone or any pyogenic surface within the substance of any hard or soft part. They possess a number of diamond-shaped openings at short distances, alternately placed at the anterior and posterior part of their circumference. In this way the fluid is drawn into the tube, and finds its way out at the extremities as soon as it is secreted.

A polypus of the œsophagus inserted into the region of the larynx, having been brought into view by a strong emetic, and seized with a *museux* forceps, and brought toward the left commissure of the mouth, a ligature was thrown round it near the base of the tongue. The tumor was then divided by the scalpel about ¾ inch in advance of the spot where the ligature had been placed. The morbid mass allowed a good deal of blood to flow, and lost color sensibly. The remains of the tumor were then pushed back, and the thread which tied it. One and the other were swallowed. The ends of the ligature were placed in the left angle of the mouth, then rolled round the ear. After 18 days the thread cut through the base of the tumor, after which the patient could eat, drink and breathe without any difficulty. The tumor measured 37 millimetres in circumference. It was concluded that it must have reached down to within two inches of the cardiac extremity of the œsophagus.—*Gazette Hebdom.*

M. Morel de Lavallée communicated to the Society of Surgeons, upon the 9th March, an observation of simultaneous luxation of the two extremities of the clavicle. The dislocation of the inner end was successfully treated, that of the outer extremity remained irreducible.

Mr. Robert reports in the *Gaz. des Hôp.* a case of phlegmonous inflammation of the cellular tissue situated under the gluteus maximus. The diagnosis was extremely difficult. After a few days, an incision, parallel to the subjacent fibres, gave exit to a large quantity of pus, and the patient was cured in about five weeks. The cellular tissue surrounding the great sciatic being engaged gave rise to most excruciating pain.

